# Synthesis and Biological Evaluation of β-Aroylpropionic acid based 1,3,4-Oxadiazoles

**DOI:** 10.4103/0250-474X.51963

**Published:** 2009

**Authors:** A. Husain, Priyanka Ahuja, Sarafroz

**Affiliations:** Department of Pharmaceutical Chemistry, Faculty of Pharmacy, Jamia Hamdard, New Delhi-110 062, India

**Keywords:** Oxadiazoles, aroylpropionic acid, antiinflammatory, analgesic, antibacterial

## Abstract

In the present investigation, two new series, 1-(4-benzylphenyl)-3-(5-substituted-1,3,4-oxadiazol-2-yl)-1-propanone and 1-(4-ethylphenyl)-3-(5-substituted-1,3,4-oxadiazol-2-yl)-1-propanone from β-(4-benzylbenzoyl)propionic acid and β-(4-ethylbenzoyl)propionic acid, respectively, were synthesized and tested for antiinflammatory, analgesic, lipid peroxidation, ulcerogenic and antibacterial actions. A fair number of compounds were found to have good antiinflammatory activity in carrageenan-induced rat paw edema test, while a few compounds showed significant antibacterial activity. The newly synthesized compounds showed very low ulcerogenic action.

A diversity of useful biological effects is possessed by heterocyclic compounds containing the five-membered oxadiazole nucleus[1]. In particular, compounds bearing 1,3,4-oxadiazole nucleus are known to exhibit unique antiedema and antiinflammatory activity[2–5]. Differently substituted oxadiazole moiety has also been found to have other interesting activities such as analgesic[3,4], antimicrobial[6,7], antitubercular[8], anticonvulsant[9] and antitumor activities[5]. The most prevalent side effects of commonly used NSAIDs are the occurrence of gastrointestinal damage with gastric upset and irritation. Studies suggest that the direct tissue contact of these agents plays an important role in the production of gastric side effects[10,11]. Aroylpropionic acids are good antiinflammatory agents but produce gastrointestinal side effects and these side effects are due to presence of free carboxylic group in the molecule[12,13]. Therefore, it was considered worthwhile to synthesize some new β-aroylpropionic acid derivatives by converting the free terminal carboxylic group into oxadiazolyl moiety with the hope to get better molecules.

As shown in Scheme 1, The starting materials, β-(4-benzylbenzoyl)propionic acid 3 and β-(4-ethylbenzoyl)propionic acid 4, were prepared by condensing diphenylmethane or ethylbenzene with succinic anhydride in presence of anhydrous aluminium chloride following Friedel-Craft's acylation reaction conditions. Reaction between β-(4-benzylbenzoyl)propionic acid 3 or β-(4-ethylbenzoyl)propionic acid 4 with aryl acid hydrazides 2a-l in phosphorous oxychloride (reaction time varies from 2 to 5 h) afforded title compounds 5a-l and 6a-e. Both analytical and spectral data [^1^H NMR, Mass (HREIMS) and IR] of the synthesized compounds are in agreement with the proposed structures.

**Scheme 1:**
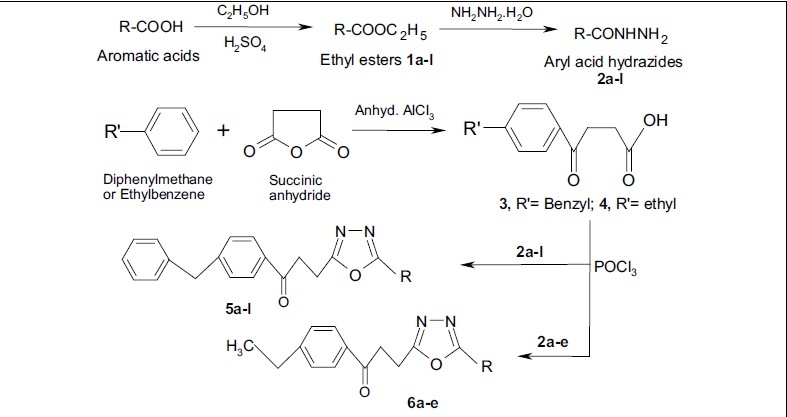
Synthetic route for the preparation of 1,3,4-oxadiazoles, 5a-l, 6a-e

Melting points were determined with the help of open glass capillaries using Kjeldahl flask containing liquid paraffin and are uncorrected. Purity of the compounds was checked by TLC on silica gel plates and spots were visualized by exposure to iodine vapors. ^1^H NMR spectra were recorded on Varian E-360 MHz or Bruker spectropsin DPX-300MHz with tetramethylsilane as internal standard in solvent CDCl_3_. The IR spectra were recorded on a Perkin-Elmer 1600 FTIR spectrophotometer in potassium bromide pellets. Mass spectra were recorded on a Jeol JMS-D 300 instrument fitted with a JMS 2000 data system at 70 eV.

Ethyl esters of aromatic acids (1a-l) and aryl acid hydrazides (2a-l) were synthesized according to the literature method[14].

Aryl hydrazide 2a (1 mol) was dissolved in phosphorous oxychloride (5 ml) and to it was added compound 3 (equimolar; 1 mmol). The reaction mixture, after refluxing for 4h, was cooled to room temperature and poured onto crushed ice. On neutralization of the contents with sodium bicarbonate solution (20%), a solid mass separated out, which was filtered and washed with water. It was crystallized from methanol to give 5a. Similarly 5b-l and 6a-e were prepared (Table 1).

**TABLE 1:** PHYSICAL CONSTANTS OF THE TITLE COMPOUNDS

Compound	R	MP (0)	Yield (%)	Molecular formula	Molecularweight
3	-	178-180	48	C_17_H_16_O_3_	268.31
4	-	110	64	C_12_H_14_O_3_	206.24
5a	C_6_H_5_	154	52	C_24_H_20_N_2_O_2_	368.43
5b	3-NO_2_ C_6_H_4_	178-180	56	C_24_H_19_N_3_O_4_	413.43
5c	4-F-C_6_H_4_	142-144	54	C_24_H_19_FN_2_O_2_	386.42
5d	4-OCH_3_-C_6_H_4_	172-174	63	C_25_H_22_N_2_O_3_	398.46
5e	C_6_H_5_-COC_6_H_4_	188	58	C_31_H_24_N_2_O_3_	472.54
5f	2-Cl-C_6_H_4_	160-162	56	C_24_H_19_ClN_2_O_2_	402.88
5g	4-Cl-C_6_H_4_	172-174	66	C_24_H_19_ClN_2_O_2_	402.88
5h	3,4-(OCH_3_)_2_-C_6_H_3_	166-168	59	C_26_H_24_N_2_O_4_	428.48
5i	C_6_H_5_-CH_2_	154	63	C_25_H_22_N_2_O_2_	382.46
5j	C_6_H_5_-OCH_2_	166	54	C_25_H_22_N_2_O_3_	398.46
5k	1-C_10_H_7_-OCH_2_	170-172	52	C_29_H_24_N_2_O_3_	448.52
5l	2-C_10_H_7_-OCH_2_	190-192	50	C_29_H_24_N_2_O_3_	448.52
6a	C_6_H_5_	122-124	62	C_19_H_18_N_2_O_2_	306.36
6b	3-NO_2_ C_6_H_4_	128-130	60	C_19_H_17_N_3_O_4_	351.36
6c	4-F-C_6_H_4_	116-118	55	C_19_H_17_FN_2_O_2_	324.35
6d	4-OCH_3_-C_6_H_4_	134-136	63	C_20_H_20_N_2_O_3_	336.38
6e	C_6_H_5_-COC_6_H_4_	154-156	57	C_26_H_22_N_2_O_3_	410.47

All the compounds were recrystallized from methanol.

In general, IR spectral data (cm^−1^) of the compounds revealed bands at 3100-3030 (C-H); 1665-1650 (C=O); 1440-1420 (C-N) and 810-750 (aromatic) and 830-815 (*p*-disubstituted). ^1^H-NMR spectral studies of the title compounds showed two triplets of two protons each at around δ 2.6 and δ 3.5 which could be assigned to two methylene protons (-CH_2_-CH_2_-). A signal was observed at around δ 4.02 in case of compounds 5a-l, which could be accounted for the methylene group (-CH_2_-) present between two phenyl rings. There appeared signals (triplet at around δ 1.26 for CH_3_CH_2_- and quartet at around δ 2.69 for CH_3_CH_2_-), for ethyl function in case of compounds 6a-e. Aromatic protons appeared in the region 7.03-8.06 ppm. Other peaks were observed at appropriate places. The mass spectra showed acylium fragments containing benzylphenyl/ethylphenyl and aryl moieties as major peaks followed by peaks with loss of CO besides the molecular ion peaks in reasonable intensities supporting the structure.

Antiinflammatory activity of the compounds was evaluated by carrageenan-induced paw edema test in rats[15], at a dose of 20 mg/kg *po*, using indomethacin as standard drug at same dose level. Swiss rats of either sex (150-200 g) were divided into control, standard, and test groups, each comprising of six rats. The protocol of animal experiments has been approved by the Institutional Animal Ethics Committee (IAEC). Freshly prepared suspension of carrageenan (0.05 ml, 1% w/v solution in 0.9% saline) was injected under the planter aponeurosis of the left hind paw of each rat. One group was kept as control and the animals of other groups were pre-treated with the test drugs suspended in 1% carboxymethylcellulose (CMC) given orally 30 min before carrageenan injection. The foot volume was measured using the mercury displacement technique, with the help of plethysmograph, both in control as well as test animals including standard drug animals, before and after 3 h of carrageenan injection. The percentage inhibition of inflammation was calculated using the formula, % inhibition = (1- Vt/Vc)×100, where, Vt and Vc are the mean relative changes in the volume of paw edema in the test and control, respectively. The results are summarised in Table 2.

**TABLE 2:** RESULTS OF ANTIINFLAMMATORY, ANALGESIC, ULCEROGENIC AND ANTIBACTERIAL ACTIVITIES

Compound	Antiinflammatory activity* (% inhibition±SEM)	Analgesic activity* (% protection ±SEM)	Ulcerogenic activity* (severity index)	Antimicrobial activity (MIC; μg/ml)	
5a	25.92±1.04	32.44±0.84	0.416	-	-
5b	19.40±1.60	24.42±0.98	0.750	>100	-
5c	33.33±1.07	19.08±0.65	0.833	50	>100
5d	52.60±0.59	40.07±0.90	0.666	>100	>100
5e	29.63±1.18	32.44±1.13	0.583	-	-
5f	36.36±1.38	51.31±0.42	0.916	>100	>100
5g	46.00±1.16	45.80±0.29	1.083	25	50
5h	56.20±2.32	54.12±0.62	0.333	-	-
5i	13.64±1.06	19.08±1.33	0.583	-	-
5j	19.35±2.20	14.33±1.50	0.666	-	>100
5k	35.60±1.28	19.08±0.65	0.833	-	-
5l	29.63±1.33	26.42±1.44	0.583	-	-
6a	25.92±2.83	24.42±1.50	0.916	-	-
6b	13.00±3.61	19.08±0.66	1.083	-	-
6c	29.63±1.54	26.42±1.44	0.833	50	>100
6d	35.60±1.96	45.12±0.11	0.750	-	-
6e	32.33±1.65	29.17±1.18	0.333	-	-
Indomethacin	64.25±2.03	nt	2.666		
Aspirin	nt	61.86±0.22	nt		
Nitrofurazone				12.5	6.5

* Number of animal used in each group is 6. ‘nt’ indicates not tested. ‘-’ indicates insignificant antimicrobial activity. SEM indicates standard error of the mean and MIC is minimum inhibitory concentration.

Analgesic activity was carried out by acetic acid induced writhing method[16] using albino mice (25-30 g) of either sex on groups of six animals each. A 1% aqueous acetic acid solution (i.p. injection; 0.1 ml) was used as writhing induced agent. Mice were kept individually in the test cage, before acetic acid injection and habituated for 30 min. Screening of analgesic activity was performed after oral administration of test drugs at the dose of 20 mg/kg. All compounds were dissolved in 1% carboxymethylcellulose (CMC) solution. One group was kept for the control experiment and received *po* administration of 1% CMC. Aspirin was used as standard at the dose of 100 mg/kg *po*. After 1 h of drug administration 0.10 ml of 1% acetic acid solution was given to mice intraperitoneally. Stretching movements consisting of arching of the back, elongation of body and extension of hind limbs were counted for 5-15 min of acetic acid injection. The analgesic activity was expressed in terms of % protection. % Analgesic activity =(*n* – *n'/n*) × 100 where *n* = mean number of writhes of control group and *n'* = mean number of writhes of test group. The percent protection in mice brought about by administration of the drugs is shown in Table 2.

Acute ulcerogenesis test was done according to Cioli *et al*[11]. Albino rats (150-200 g) were divided into different groups consisting of six animals in each group. Ulcerogenic activity was evaluated after oral administration of test compounds or indomethacin at the dose of 60 mg/kg. Control rats received oral administration of vehicle (suspension of 1% methylcellulose). Food but not water was removed 24 h before administration of the test compounds. After the drug treatment, the rats were fed normal diet for 17 h and then sacrificed. The stomach was removed and opened along the greater curvature, washed with distilled water and cleaned gently by dipping in saline. The gastric mucosa of the rats was examined by means of a magnifying glass. For each stomach, the severity of mucosal damage was assessed according to the following scoring system: 0.5- redness; 1.0- spot ulcers; 1.5- hemorrhagic streaks; 2.0- ulcers<3, but ≤5; 3.0- ulcers>5. The mean score of each treated group minus the mean score of the control group was considered as severity index of gastric mucosal damage.

Antibacterial activity of newly synthesized compounds was determined against the bacterial strains gram positive (*Staphylococcus aureus;* NCTC-6571) and gram negative (*Escherichia coli;* ATCC-25922). The test was carried out according to the turbidity method[17]. A solution of the compounds was prepared in dimethylformamide (DMF) and a series of doubling dilutions prepared with sterile pipettes. To each of a series of sterile stoppered test tubes a standard volume of nutrient broth medium was added. A control tube containing no antimicrobial agent was included. The inoculum consisting of an overnight broth culture of microorganisms was added to separate tubes. The tubes were incubated at 37°; for 24 h and examined for turbidity. The tube with highest dilution showing no turbidity was MIC. Nitrofurazone was used as standard drug.

The antiinflammatory activity (Table 2), revealed that the compounds 5d and 5h showed very good antiinflammatory activity (52.60 and 56.20% inhibition, respectively), which was comparable to that of indomethacin (64.25% inhibition). Amongst the compounds subjected to analgesic activity (Table 2), compounds 5f and 5h were found to possess significant activity (51.31 and 54.12% protection, respectively), while the standard drug aspirin showed 61.86% activity. All the synthesized compounds showed very low ulcerogenic activity (0.416-1.083 severity index), whereas the standard drug indomethacin showed high severity index of 2.666 (Table 2). The results indicate that the compounds are almost devoid of ulcerogenic action. From the antibacterial results (Table 2), it was observed that the compound 5g was the most active among the tested compounds with MIC- 25 μg/ml against *S. aureus* and MIC- 50 μg/ml against *E. coli*. The rest of the compounds were moderate or inactive in their action.
